# Activity Following Total Hip Arthroplasty: Which Patients Are Active, and Is Being Active Safe?

**DOI:** 10.3390/jcm12206482

**Published:** 2023-10-12

**Authors:** Laura Elisa Streck, Yu-Fen Chiu, Sebastian Braun, Anisa Mujaj, Carola Hanreich, Friedrich Boettner

**Affiliations:** 1 Adult Reconstruction and Joint Replacement Department, Hospital for Special Surgery, 535 East 70th Street, New York, NY 10021, USA; 2 Biostats Core, Research Administration, Hospital for Special Surgery, 535 East 70th Street, New York, NY 10021, USA; 3 Center for Musculoskeletal Surgery, Charité Universitätsmedizin Berlin, Member of Freie Universität Berlin and Humboldt University Berlin, Chariteplatz 1, 10117 Berlin, Germany; 4 Department for Visceral Surgery and Medicine, Inselspital, Bern University Hospital, University of Bern, Freiburgstrasse, 3010 Bern, Switzerland

**Keywords:** outcome, activity, revision, hip replacement, sports, lower extremity activity scale

## Abstract

Background: Younger and physically active patients demand a return to sport after total hip arthroplasty (THA). However, because of the risk of implant wear and loosening, high-impact activities are often not recommended. The current study evaluates predictive factors and revision rates in patients with higher activity levels. Methods: This retrospective study included 4152 hips in 3828 patients aged 45–75 that underwent primary THA for primary osteoarthritis between 2009 and 2019 with a minimum follow-up of 2 years. Pain and Lower Extremity Activity Scale (LEAS) were assessed before and 2 years after surgery. Activity was classified as low (LEAS 1–6), moderate (LEAS 7–13), or high (LEAS 14–18). Results: Pain and LEAS improved from preoperative to 2-years postoperative (*p* < 0.001). The activity level was low in 6.2%, moderate in 52.9%, and high in 40.9% of the patients. Younger age, lower BMI, ASA, and CCI, male sex, and higher preoperative LEAS correlated with higher activity at 2 years (*p* < 0.001). The predicted revision-free survival rates between the activity groups were better for more highly active patients (*p* < 0.001). Conclusions: High physical activity 2 years following THA, with participating in sports like jogging several times a week, did not increase the risk of revision surgery. THA patients should not be prevented from a highly active lifestyle.

## 1. Introduction

Physical activity following total hip arthroplasty (THA) is generally recommended, since being active not only improves cardiovascular and general health but also improves bone quality and therefore implant fixation. Increased physical activity has also been associated with reduced risk of falls [1,2,3,4]. However, most specialty societies recommend against higher-impact sports.

Wear in THA has been reported as a function of use, and higher activity has been associated with higher THA revision rates [5,6]. Surveys amongst members of the hip society in both 1999 and 2005 revealed that surgeons did advise their patients against sports like jogging [7]. However, there have been relevant changes in materials and implant designs over the past years. In particular, the introduction of highly cross-linked polyethylene has significantly reduced THA revision rates [8,9,10]. At the same time, younger and more active patients today demand a return to a more active lifestyle. In addition, the number of young patients undergoing THA is increasing [11]. These patients have higher expectations, and the majority of previously active patients desire to return “back to normal” regarding their sports activities [12,13,14].

Studies focusing on sports like skiing, which are linked to higher implant wear rates did not show higher revision rates. Furthermore, habitual jogging was not associated with adverse effects [15,16,17]. Comparing highly active versus less active patients, an evaluation of more than 2500 THA patients with a follow up of 4.5 years reported that high activity improved implant survival, even after adjusting for potential confounding factors such age, gender, or body mass index (BMI) [18]. 

Recent surveys among arthroplasty surgeons showed that recommendations regarding sports activities following THA are becoming less restrictive. However, full-contact sports such as soccer and martial arts have mostly remained prohibited for THA patients [19,20]. The current literature mainly focusses on either the influence of certain specific sports or on single complications such as dislocations [15,16,17,21]. Studies investigating overall revision rates mostly compare active versus inactive patients or younger versus older patients [18,22].

The current study aims to answer the following research questions: (1) Is there a difference in THA revision rates between low, moderate, and highly active patients? (2) Which patient-specific factors affect activity two years after THA? 

## 2. Materials and Methods

The study received approval by the authors’ institutional review board. Patients were included consecutively if they met the following criteria: (1) primary THA between 2009 and 2019 at the authors’ institution, (2) primary osteoarthritis of the hip as indication for surgery, (3) age between 45 and 75 years, and (4) minimum follow-up of 2 years. A total of 4152 hips in 3828 patients were available for evaluation (39.1% males, 47.0% left hips). Bearing types were ceramic head and polyethylene (PE) liner in 62.6%, metal head and PE liner in 37.0%, and ceramic on ceramic in 0.4%. Overall, 16.0% of the implants were dual-mobility implants. All patients underwent the same postoperative protocol including prescriptions for physical therapy. Patients followed postoperative hip precautions for 4 weeks after surgery. They were advised to avoid end-range flexion, adduction, and internal rotation (posterior approach) as well as end-range extension and external rotation (anterior approach). 

### 2.1. Outcome Parameters

Pain and activity level were assessed prior to THA and at a 2-year follow-up. Pain was measured on a numeric rating scale (NRS) from 0 “no pain” to 100 “worst pain”. Activity was measured using the Lower Extremity Activity Scale (LEAS) [23]. Patients were grouped into low activity (LEAS 1–6, 256 hips, 6.2%), moderate activity (LEAS 7–13, 2196 hips, 52.9%), and high activity (LEAS 14–18, 1700 hips, 40.9%). Age, BMI, Charlson comorbidity index (CCI) and American Society of Anesthesiology score (ASA) were assessed at the time of primary surgery. Revision surgeries were assessed by retrospective chart review.

### 2.2. Data Collection

All data were collected retrospectively from the patients’ medical record system at the authors’ institution in line with the inclusion and exclusion criteria. Pain measured on an NRS and LEAS is a regular part of the preoperative patient assessment and of the 2-year follow-up. Data were either collected by email questionnaire or at the time of the 2-year follow-up visit.

### 2.3. Statistical Analysis

Descriptive analysis was reported as mean and standard deviation (SD) for continuous variables, and frequency and percentage for categorial variables. Cohorts were compared with the Chi-square -test for categorial variables and analysis of variance (ANOVA) with multiple comparisons for continuous variables. The Pearson correlation coefficient (r) was calculated to determine the strength of the correlation. Preoperative and postoperative NRS and LEAS were compared using a paired *t*-test. A Kaplan–Meier curve was plotted to predict implant survival, and differences in implant survival between the activity groups were calculated with the log-rank test. Logistic regression was performed to assess correlation between LEAS at the 2-year follow-up and predicted implant survival. Odds ratio (OR) and 95% confidence intervals (CI) were reported from the logistic regression analysis. OR was then converted to predicted probability. A Cox proportional hazard model was used to adjust for confounding variables (Age, BMI, Sex, CCI, ASA) when comparing the outcome variable “time to revision” between activity groups. All analyses were two-tailored. Statistical testing was performed for a significance-level of alpha = 0.05. Data analysis was conducted using SAS 9.4 (SAS Institute Inc., Cary, NC, USA).

## 3. Results

### 3.1. Demographics

Table 1 depicts preoperative demographic information of the study cohort. The reduction in pain from preoperative to 2-years postoperative was significant, with a mean reduction of 54 points on the NRS (SD 27, *p* < 0.001). Function significantly improved from preoperative to 2-years postoperative, and the mean difference in LEAS was 1.7 points (SD 3.6, *p* < 0.001).

### 3.2. Activity Groups

**Figure 1:**
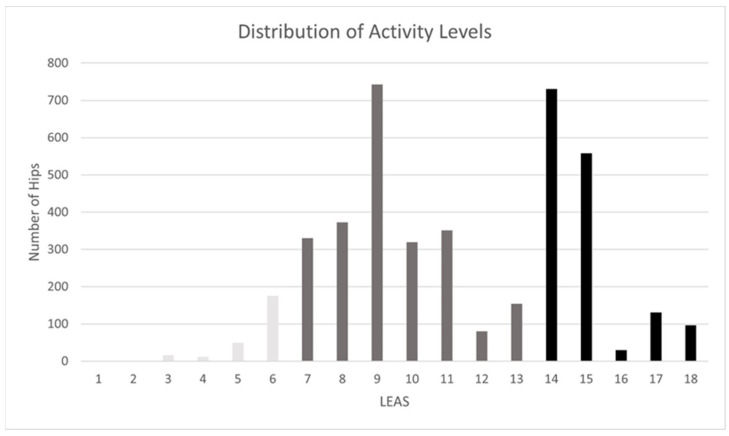
Distribution of the activity levels according to LEAS score 2 years after THA implantation. Low activity was defined as LEAS 1–6 (light grey), moderate activity as LEAS 7–13 (dark grey), and high activity as LEAS 14–18 (black).

At the 2-year follow-up, 6.2% of the cases showed low activity, 52.9% moderate activity, and 40.9% high activity. Figure 1 displays the distribution of LEAS levels amongst the study group in detail. Age (r = −0.263, *p* < 0.001) and BMI (r = −0.208, *p* < 0.001) were negatively correlated with activity level. Higher pain scores at the 2-year follow-up correlated with lower LEAS (r = −2.4, *p* < 0.001). A higher preoperative LEAS correlated positively with postoperative LEAS (r = 0.381, *p* < 0.001). Male sex, lower ASA, and lower CCI were significantly associated with higher activity (*p* < 0.001, respectively).

### 3.3. Revisions

**Figure 2:**
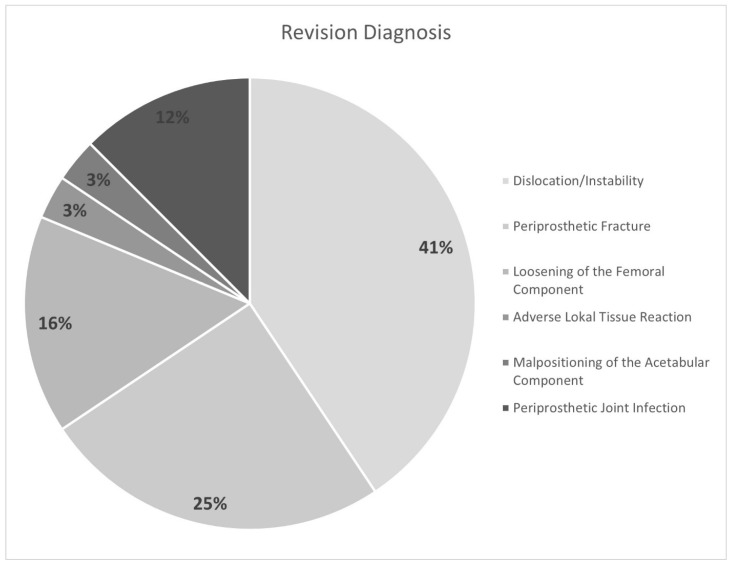
Indications for revision surgery, as percentage of all revisions.

Predicted revision-free survival was 99.7% at 2 years, 98.8% at 5 years, and 97.3% at 7 years. A total of 32 hips (0.8%) underwent revision surgery with component exchange. The mean time from implantation to revision was 26 months (range 0–67 months, SD 22 months). Indications for revision are shown in Figure 2. The most common diagnosis was dislocation/instability (13), followed by periprosthetic fracture (8), loosening of the femoral component (5), periprosthetic joint infection (PJI, 4), adverse local tissue reaction (ALTR, 1), and malpositioning of the acetabular component (1). The mean time to revision in the low-activity groups was 20.1 months (range 0–50, SD 18.2), in the moderate-activity group 30.6 months (range 0–67, SD 23.0), and in the high-activity group 21.6 months (range 0–64, SD 21.9).

**Figure 3:**
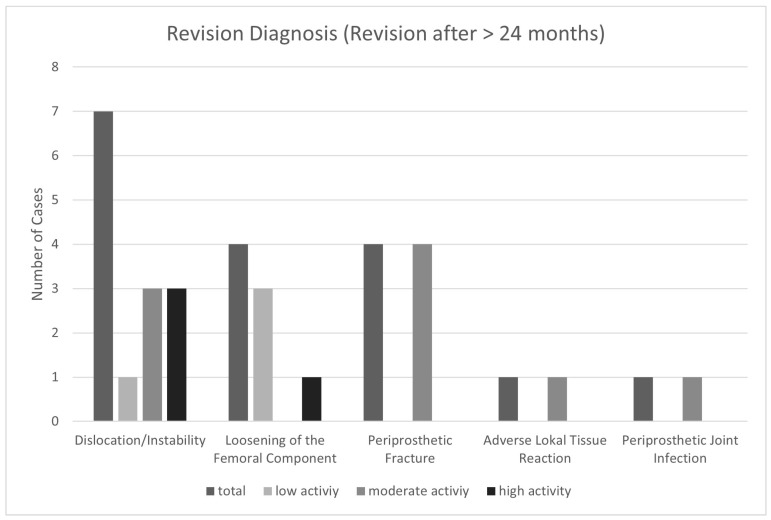
Indications for revisions that occurred >24 months following primary THA implantation grouped by the patients’ activity level.

A total of 15 of the 32 revision cases underwent revision within 2 years following primary implantation and were therefore excluded from the correlations between activity levels (assessed at 2 years) and revisions. The indications for revision after 24 months and the distribution based on the activity groups is presented in Figure 3.

**Figure 4:**
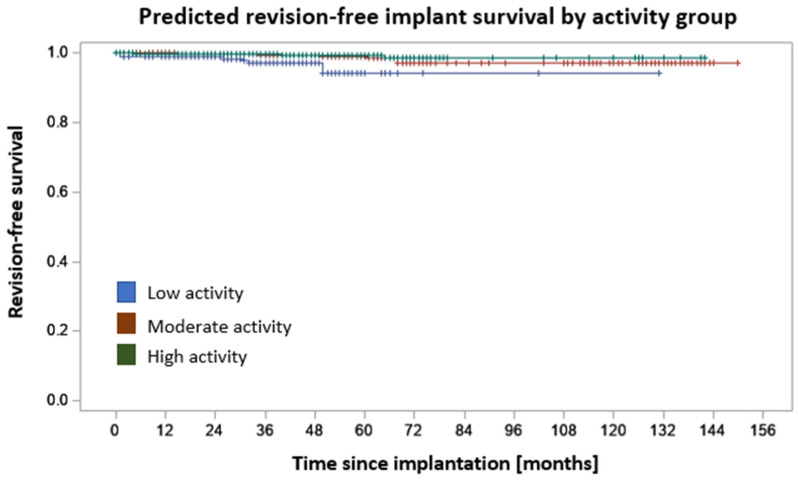
Kaplan–Meier curve on predicted revision-free survival grouped by activity level. For details on survival rates and comparison of the groups, please see the main manuscript.

The predicted survival rates between the activity groups differed significantly (*p* < 0.001), with favorable results for the high-activity patients. The Kaplan–Meier curve on revision-free survival for the three activity groups, respectively, is presented in Figure 4. The 5-year revision-free survival in the low-activity group was 94.2%, in the moderate-activity group 98.8%, and in the high-activity group 99.3%. The 7-year revision-free survival rates were 94.2%, 96.9%, and 98.4%, respectively. After adjusting for potential confounding variables (Age, BMI, Sex, ASA, CCI), the low-activity group still showed a higher risk of revision compared to the moderate-activity group (HR 0.17, *p* = 0.002) and the high-activity group (HR 0.11, *p* < 0.001).

## 4. Discussion

THA is a highly successful surgery, and the current study showed significant improvement in both function measured with LEAS and pain from preoperative to 2-years postoperative [14]. The mean LEAS was 11.2 points, which is comparable to previous results from Bauman et al., who reported a mean University of California, Los Angeles score (UCLA) of 6, 1 year after THA [24].

Which activity level is achieved following THA is influenced by various factors. The current study showed that better overall health and fewer comorbidities (both according to ASA score and CCI), as well as a higher preoperative level of activity (LEAS), were significantly associated with higher activity (*p* < 0.001), which is in line with previous findings [25,26]. Reports on the association between female sex and older age with less activity are controversial in the literature [17,25,27,28,29,30]. However, our data showed a significant correlation (*p* < 0.001, respectively) for both factors. 

Other than most patient-specific factors, the BMI is modifiable and offers opportunity for active intervention. A higher BMI has not only shown to increase the risk of complications, especially infections, following THA [31,32]; it was also significantly associated with lower activity in the current study cohort as well as in previous studies [25,28]. To reduce complications and achieve higher activity following THA, preoperative weight reduction in overweight patients may be beneficial.

In the current study, at 2-years postoperative, the majority of patients (53%) participated in moderate activity, defined as activities like walking outside at least one to two blocks at a time to participating occasionally in physical activities like jogging or dancing. Overall, 40% achieved a high level of activity, at a minimum participating in sports such as jogging several times a week. 

The comparison of studies on “activity” and “sports” after THA is problematic due to the inconsistent definition of these terms. Ollivier et al. defined sporting activity as University of California Los Angeles score (UCLA) > 5 and reported a return to sports (RTS) rate of 64% after a mean follow-up of 9.8 years [33]. Huch et al. reported that the rate of patients participating in defined sporting activities at least 1 h a week increased from 36% preoperatively to 52% at a 5-year follow-up [27]. Bonnin et al. followed up on 1206 hips and distinguished between light, moderate, and strenuous sports, and found that 73% did at least one moderate sport and 20% did at least one strenuous sport [28]. Overall, both the current study as well as the literature suggest that the majority of patients are able to improve their physical activity and return to sports following THA. 

Investigating reasons for inactivity in THA patients, Delasotta et al. looked at a cohort of THA patients younger than 50 years old and found that 26% of patients that had stopped physical activities did so because of the physicians’ recommendations [34]. A meta-analysis by Ollivier et al. found physicians’ recommendations to be the reason for not participating in sports activities in 17% of the patients [33]. Arshi et al. reported that activity restrictions were more commonly self-imposed than due to the surgeons’ recommendations [25].

However, while most surgeons generally recommend physical activity, a survey amongst arthroplasty surgeons in 2020 showed that 34% did not recommend high-impact sports [19]. However, there is no consensus on which sports should be considered “high-impact”. The joint load during certain movements highly depends on the speed with which the movement is carried out [35,36]. Van den Bogert et al. found lower joint-contact forces during skiing with long turns on a flat slope compared to running at 3.5 m/s. However, skiing with short turns on a steep sloped showed far higher contact forces [36]. Bowling was regularly considered a recommended low-impact sport, yet when evaluating joint contact force and torsion torque in working-age THA patients, Bender et al. found higher forces on the hip during bowling than during high-impact soccer [37,38]. They also found that working-age patients showed higher contact loads compared to retirement-age patients during daily activities. Based on these results, it has been suggested that recommendations of sporting activities should be mainly focused on low-impact activities, especially for younger patients [37]. Higher revision rates for younger patients and males compared to females have been reported previously. It has been hypothesized that this may be due to higher activity of these patient populations [39]. Furthermore, it has been shown that polyethylene wear in THA is a function of use, not time [6]. However, more recent studies showed that new materials developed over the past decades are likely to reduce this impact [8,9].

Nevertheless, there are several risks associated with physical activities. They may depend on various interrelated aspects such as the patient’s prior experience and level at the sport, the frequency of the performance, the agility, range of motion, and risk of falls or uncontrolled movement. The individual bone stock may influence the risk of fractures. The current study showed that the predicted revision-free survival differed significantly between the activity groups, with the best results for highly active patients. It may be hypothesized that more active patients are younger and have a better bone stock. Yet after adjusting the groups for age, BMI, CCI, ASA, and preoperative activity, the low-activity group still showed inferior revision-free survival compared to the moderate- as well as the high-activity group. Similar findings were reported by Crawford et al. in 2021, who found that a higher activity level (defined as UCLA > 6) was a significant factor for improved implant survival after THA [18]. 

The most common reasons for revision after 24 months in the current cohort were dislocation, fracture, and loosening. While the absolute number of dislocations in the moderate- and high-activity group (three, respectively) was higher than in the low active group (one dislocation), the percentage was still higher for the latter. This is in line with van der Weegen et al., who found that less restrictions and precautions did not lead to worse dislocation rates following THA [21]. In the current cohort, all periprosthetic fractures requiring revision occurred in the moderate-activity group. It may be hypothesized that a high level of activity may improve proprioception and the ability to catch yourself in a fall. However, case numbers were too low to draw evidence-based conclusions on this from the current data.

Aseptic loosening occurred in three low-activity patients and only one high-activity patient. The risk of aseptic loosening due to implant wear is frequently cited as an argument against high activity [5,6,19,37]. However, the current findings suggest that, despite the biomechanical proof of increased wear due to increased forces, actual clinical findings may not justify general advice against high physical activity [17,18]. The risk associated with being highly active in general and participating in certain desired sports specifically should be evaluated and discussed together with the patient on an individual basis rather than imposing generalized restrictions. The current study showed that being highly active and participating in sports like jogging at least several times a week did not increase the risk of undergoing revision surgery on a mid-term follow-up. However, data on long-term results are limited and further studies are needed to gain more evidence. 

There are limitations to this study: (1) it cannot be ruled out that patients were less active at 2-years postoperative because problems already existed that may have led to a later revision; (2) additional confounding factors might influence outcomes following THA; (3) different bearings and surgical approaches to the hip were included in the current study; (4) the case numbers varied between the activity groups; (5) the fact that the revision rate was not increased at 2 years might not predict that higher activity sports are save in long term follow ups; (6) as patients with secondary osteoarthritis as well as very young and older patients were excluded from the study, the current results may not apply to these patient groups; (7) this was a single-center study and all patients were operated on by specialized high-volume arthroplasty surgeons, which may add to low revision rates.

## 5. Conclusions

Younger age, lower BMI, male sex, lower ASA and CCI score, and higher preoperative activity were predictors for higher postoperative activity levels. A total of 40% of patients achieved a high level of activity 2 years after surgery, participating in sports like jogging at least several times a week. Higher levels of activity did not increase the risk of undergoing subsequent revision surgery, and the current data support an active lifestyle following THA. However, further studies are necessary to provide long-term follow-up data.

## Figures and Tables

**Table 1:** Demographics of all patients at the time of THA implantation.

	Mean	SD	%
Age (years)	64	10	
BMI (kg/m^2^)	28.4	5.9	
ASA	N/A	N/A	
1			4.9
2			79.3
3+			14.9
CCI	N/A	N/A	
0			71.3
1–2			25.8
3+			2.7
LEAS (points)	9.5	3.2	
Pain (NRS)	61	24	

## Data Availability

The data presented in this study are available on reasonable request from the corresponding author. The data are not publicly available due to ethical restrictions.
